# Exploring the Impact of Organizational Identification on Innovative Work Behavior in the Korean Public Sector: The Moderating Role of Charismatic Leadership

**DOI:** 10.3390/bs15091218

**Published:** 2025-09-08

**Authors:** Kuk-Kyoung Moon, Jaeyoung Lim

**Affiliations:** 1 Department of Public Administration, Inha University, Incheon 22212, Republic of Korea; kkmoon@inha.ac.kr; 2 Department of Public Administration and Social Welfare, Chosun University, Gwangju 61452, Republic of Korea

**Keywords:** organizational identification, charismatic leadership, innovative work behavior

## Abstract

Public sector organizations increasingly face demands for innovation. However, the hierarchical and rule-bound nature of bureaucracy can hinder employees’ ability to engage in creative and change-oriented behavior. This study investigates how organizational identification—a psychological sense of oneness with the organization—is associated with innovative work behavior among South Korean public officials and how this relationship is moderated by charismatic leadership. Grounded in social identity theory and leadership literature, we argue that employees with strong organizational identification are more likely to exhibit innovative work behavior, particularly when supported by leaders who articulate an inspiring vision and embody core public values. Using data from the 2024 Comparative Survey on Perceptions of Public and Private Sector Employees (N = 1012), hierarchical regression analyses reveal that both organizational identification and charismatic leadership significantly promote innovative work behavior. Furthermore, charismatic leadership enhances the positive effect of identification on innovative work behavior. These findings contribute to behavioral public administration research by clarifying how employee identity and leadership style interact to foster innovation, even within rigid procedural environments.

## 1. Introduction

Public organizations today face intense pressure to innovate in response to complex societal challenges and rising public expectations (Lindquist, 2022). Governments are urged to devise creative solutions for issues ranging from digital service delivery to climate resilience; yet, bureaucratic structures often impede such innovation (Fleischer & Wanckel, 2024). Rigid hierarchies, formal rules, and risk-averse cultures can stifle employees’ creativity and adaptability (Criado et al., 2025). Consequently, a persistent challenge in the public sector is unlocking innovative work behavior at the individual level, despite the inertia inherent in bureaucratic rigidity (Cinar et al., 2021). This dilemma highlights the need to understand what motivates public employees to generate and implement new ideas within traditionally inflexible organizations.

A promising motivator of innovative work behavior in the public sector is organizational identification—the degree to which individuals internalize their organization’s values and perceive themselves as integral members of it (Yang & Mostafa, 2024). Drawing on social identity theory, employees who view their organization as a core part of their self-concept are more likely to align their behavior with organizational goals and invest effort into its success (Ellemers et al., 2004). In this context, identification fosters a sense of shared purpose that drives individuals to exceed formal duties and engage in proactive, improvement-oriented actions (Srirahayu et al., 2023). While much existing literature has concentrated on public service motivation or organizational culture as drivers of innovation (Miao et al., 2018; Wynen et al., 2017), the role of identification remains relatively underexplored. In environments where external incentives are limited and bureaucratic constraints prevail, a robust psychological attachment to the organization can serve as a significant intrinsic motivator. This study aims to address this gap by examining how organizational identification contributes to innovative work behavior in public organizations.

Although employee identification provides a psychological basis for innovation, this potential does not automatically translate into tangible innovative work behavior (G. Zhang & Wang, 2022). For organizational identification to lead to innovative work behavior, a contextual condition is often necessary—one that reinforces employees’ shared sense of purpose and aligns their psychological attachment with organizational goals. Charismatic leadership fulfills this role by fostering emotional and motivational alignment with the organization’s mission. Through visionary communication, symbolic actions, and confidence in followers’ capabilities, charismatic leaders frame innovation as a collective pursuit rather than an individual risk (Meslec et al., 2020; Tavares et al., 2021). This is particularly crucial in the public sector, where hierarchical rigidity and risk aversion can suppress creative behavior. For employees with strong organizational identification, charismatic leadership transforms passive alignment into proactive engagement. It elevates innovation to an authentic expression of shared values and institutional purpose, thereby enhancing its moral and emotional resonance (Conger & Kanungo, 1998). This study is the first to empirically test the interaction between charismatic leadership and organizational identification, demonstrating how charisma amplifies the identity-based pathway to employee innovative work behavior in public organizations.

This research is situated in the context of South Korean public organizations, which are characterized by strong hierarchical structures, collectivist cultural norms, and centralized administrative control. These features provide a theoretically meaningful boundary condition for understanding the proposed relationships: in such environments, employees may be especially responsive to value-based, emotionally resonant leadership that aligns with their internalized organizational identity. Rather than viewing the Korean setting as a sampling limitation, this study conceptualizes it as a context where the synergy between charismatic leadership and organizational identification is particularly salient—thus offering insights that are transferable to similarly structured bureaucratic systems.

**Figure 1:**
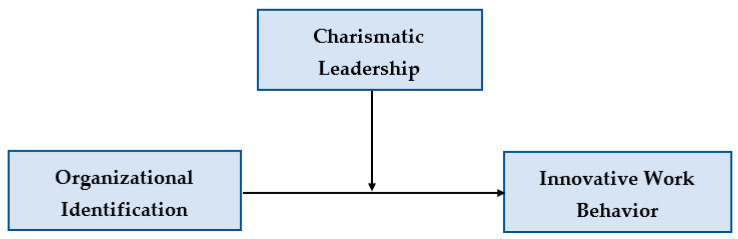
Hypothesized model.

Based on this theoretical framework, this study presents the research model shown in Figure 1. The primary objective is to examine how organizational identification relates to innovative work behavior among public sector employees and to assess whether charismatic leadership moderates this relationship. In the model, organizational identification is the independent variable, innovative work behavior is the dependent variable, and charismatic leadership is the moderating variable that may strengthen the positive effect of identification on innovation.

This study contributes to the behavioral public administration literature by examining the conditions under which organizational identification translates into innovative work behavior in bureaucratic environments. While previous research has treated employee motivation and leadership as separate influences on innovation, our approach integrates the two by theorizing that organizational identification provides intrinsic motivation, though its behavioral consequences depend on contextual activation. Drawing on social identity theory, we argue that identification alone serves as a latent motivational resource. Building on leadership theory, we conceptualize charismatic leadership as the mechanism that activates this latent resource by embodying institutional values, inspiring confidence, and fostering a climate where risk-taking is encouraged. Thus, charismatic leadership acts as a contextual force that determines whether identification remains symbolic or evolves into innovative action.

Using large-scale survey data from public organizations in South Korea, this study explores how internal motivation and leadership dynamics jointly foster innovation among government employees. Although situated in a specific national context, the findings provide broader insights for public organizations operating in rule-bound or structurally rigid environments. By highlighting the interplay between psychological attachment and leadership conditions, this study elucidates how innovative work behavior can emerge even within traditionally formalized and hierarchical systems.

The rest of this article is organized as follows. The next section reviews the literature on organizational identification, innovative work behavior, and charismatic leadership while developing research hypotheses. The third section outlines the research methods, including data sources, measurement, and analytical procedures. The fourth section presents the results of empirical analysis. The final section discusses the implications of the findings and the study’s theoretical and practical contributions.

## 2. Theories and Hypothesis Development

### 2.1. The Impact of Organizational Identification on Innovative Work Behavior

Innovative work behavior involves the intentional creation, promotion, and implementation of new ideas within a role, group, or organization, aimed at enhancing products, services, or work processes (Janssen, 2000). This behavior encompasses a range of activities, including identifying problems, generating ideas, advocating for those ideas, and executing them (De Jong & Den Hartog, 2010). It is widely viewed as essential for organizational adaptability, effectiveness, and long-term success in ever-changing environments (Messmann & Mulder, 2012; Srirahayu et al., 2023). While innovative work behavior has often been highlighted in the private sector due to competitive pressures and profit motives, it is equally, if not more, crucial in the public sector (De Vries et al., 2016). Today, government agencies face rapidly evolving technological landscapes, increasing citizen expectations, and complex societal challenges (Mergel et al., 2019). Addressing issues such as digital transformation, climate resilience, and social inequality necessitates not only administrative skills but also the creative and proactive involvement of public employees (Lee & Kim, 2024). However, public organizations typically lack the extrinsic incentives prevalent in private firms and operate within rigid bureaucratic structures, political oversight, and limited resources (Borins, 2001; Kroll & Porumbescu, 2019). Therefore, fostering innovative work behavior in these contexts requires leveraging intrinsic motivation.

A key driver of innovative work behavior in public organizations is organizational identification—an employee’s psychological attachment and sense of belonging to their organization (Yang & Mostafa, 2024). When individuals identify with their organization, they tend to internalize its mission and values, which enhances intrinsic motivation to exceed formal job expectations (Carmeli & Freund, 2009; Riketta, 2005). This connection encourages discretionary behaviors such as proposing improvements, initiating change, and supporting colleagues—all fundamental aspects of innovative work behavior. In bureaucratic environments with limited extrinsic rewards, this form of identification becomes particularly beneficial (Wright & Pandey, 2011). Employees who view organizational goals and successes as their own are more likely to feel a sense of ownership and responsibility, motivating them to engage in proactive problem-solving and service enhancements (Van Knippenberg, 2000). Thus, organizational identification not only boosts employee well-being and commitment but also strategically fosters innovation within public institutions.

This relationship can be understood through social identity theory, which suggests that individuals derive part of their self-concept from their group affiliations, such as their organizational membership (Ashforth & Mael, 1989). When employees see themselves as integral members of their organization, they align their behaviors with collective interests and cultivate a sense of shared purpose and efficacy (Ellemers et al., 2004). In public service contexts, this identification serves as a strong source of intrinsic motivation, as employees perceive organizational success as a reflection of their own self-worth (Irshad & Bashir, 2020). Consequently, those with strong organizational identification are more likely to challenge the status quo, propose creative solutions, and embrace the risks associated with innovation. Their professional identity drives them to take initiative, viewing their contributions as meaningful to the collective mission (Lee & Kim, 2024; Srirahayu et al., 2023; G. Zhang & Wang, 2022).

Empirical evidence supports this perspective: research indicates that organizational identification is positively related to employees’ innovative work behavior. For instance, Shim and Faerman (2017) found that organizational identification is positively associated with organizational citizenship behavior among public employees in South Korea. Employees with a strong sense of belonging are more likely to engage in cooperative and discretionary actions. Rho et al. (2015) demonstrated that organizational identification significantly increases extra-role behavior and reduces voluntary absenteeism among state government employees in Georgia and Illinois. Employees who strongly identify with their organization are more likely to participate in discretionary, cooperative actions and less likely to miss work without valid reasons. These findings underscore the motivational role of identification in influencing both positive and negative employee behaviors. Karolidis and Vouzas (2019) studied Greek public sector employees and found that organizational identification significantly enhances helping behavior, indicating that when public servants strongly identify with their organization, they are more willing to support colleagues and contribute to a collaborative work environment.

In summary, when organizational identification is high, public employees often exceed formal job expectations to promote the organization’s best interests, including championing new ideas and adopting innovative practices to improve public service outcomes. To validate this proposition, the following hypothesis will be tested:

**Hypothesis** **1:** *Organizational identification is positively associated with innovative work behavior among public sector employees.*

### 2.2. Moderating Role of Charismatic Leadership

The strength of the relationship between organizational identification and innovative work behavior can vary significantly based on contextual and situational factors (Y. Liu et al., 2011). According to contingency theory, this relationship is not uniform but is moderated by external elements, particularly leadership style, which can either enhance or inhibit the expression of identification (Koeswayo et al., 2024; Seong & Hong, 2018). Importantly, organizational identification does not automatically produce innovation, especially in bureaucratic environments where hierarchical norms and risk aversion prevail. Its translation into innovative action requires leaders who provide legitimacy and psychological safety, ensuring that employees feel authorized and secure to act on their identification. When leadership fosters a strong sense of shared purpose and affirms employees’ value to the organization, those with high identification may be more motivated to act innovatively (Goswami et al., 2018; Y. Liu et al., 2019). Conversely, leadership that lacks emotional resonance or meaningful direction may weaken this relationship. These dynamics suggest that leadership style influences the strength of the connection between organizational identification and innovative work behavior.

A critical situational factor is leadership style, especially charismatic leadership, which refers to a leader’s ability to inspire and mobilize followers through a compelling vision and personal influence (Meslec et al., 2020). Charismatic leaders convey emotionally resonant messages, articulate visionary goals aligned with shared values, and engage in symbolic actions that build trust and commitment (Paulsen et al., 2009; Shamir & Howell, 1999). They are sensitive to both organizational contexts and followers’ psychological needs, allowing them to tailor their approach to foster emotional engagement (Conger & Kanungo, 1998). Charismatic leaders often take personal risks, challenge the status quo, and exhibit the courage to pursue meaningful change—actions that energize followers and legitimize innovation (Gebert et al., 2016; Javidan & Waldman, 2003). They also utilize rhetorical strategies, such as metaphors and emotionally charged narratives, to frame organizational challenges as collective missions requiring joint efforts (Meslec et al., 2020). Ultimately, by embodying institutional values and serving as symbolic leaders, charismatic leaders reinforce collective identity and act as focal points for coordinated, purpose-driven behavior (Conger & Kanungo, 1998).

While transformational, servant, and ethical leadership approaches all emphasize values and follower development, charismatic leadership offers distinct advantages in bureaucratic environments constrained by rigid rules and hierarchical norms (Hameduddin & Engbers, 2022; Schwarz et al., 2020). Unlike transformational leadership, which focuses on rational goal alignment and systemic reform, charismatic leadership energizes employees through emotionally charged vision framing and symbolic communication, providing not only strategic direction but also affective momentum (Tavares et al., 2021). Although often conceptualized as a sub-dimension of transformational leadership—specifically as idealized influence—charisma merits theoretical distinction in this study (Levine et al., 2010). Its emotionally resonant and identity-activating mechanisms are particularly effective in cultures with high power distance and normative conformity, such as the Korean public sector, where conventional structural levers for change are limited (Kim & Song, 2011). While servant and ethical leadership are grounded in moral principles and crucial for building trust, they tend to prioritize stability and fairness over disruption. In contrast, charismatic leadership legitimizes selective rule-breaking for higher institutional purposes, embedding innovation within the organization’s identity narrative (Babcock-Roberson & Strickland, 2010). Through rhetorical dramatization, moral conviction, and symbolic action, charismatic leaders may foster psychological safety for risk-taking and activate a shared identity as a driver of innovation—making charisma uniquely relevant in overcoming bureaucratic inertia.

Charismatic leadership serves as a powerful contextual enhancer that strengthens the relationship between organizational identification and innovative work behavior. First, charismatic leaders enhance psychological safety by expressing confidence in employees’ abilities, reducing the perceived risk of failure, and normalizing creative experimentation. Second, they provide legitimacy for risk-taking by modeling bold behavior, articulating a moral justification for change, and aligning innovation with core organizational values. Third, they boost creative self-efficacy by setting high expectations, offering individualized support, and celebrating incremental successes—thereby reinforcing employees’ belief in their capacity to innovate. In this context, employees view innovation as a meaningful contribution to shared goals rather than merely a technical task (Paulsen et al., 2009). For employees who already strongly identify with their organization, this vision acts as a motivational multiplier, reinforcing their willingness to take initiative for the collective good (Javidan & Waldman, 2003). Additionally, charismatic leaders help diminish psychological barriers to innovation—such as ambiguity, perceived risk, and fear of failure—by setting high yet attainable expectations, expressing confidence in their followers’ abilities, and actively supporting creative endeavors (Le Blanc et al., 2021). This supportive climate empowers highly identified employees to direct their loyalty toward novel ideas and unconventional solutions. Furthermore, by modeling behaviors that exemplify core institutional ideals, charismatic leaders foster a deeper alignment between organizational goals and personal values, enabling employees to translate their abstract loyalty into meaningful, identity-driven actions (Antonakis et al., 2016; Chang, 2018; Yan et al., 2025). Moreover, charismatic leaders stimulate intellectual engagement by encouraging critical thinking and challenging established routines, fostering a dynamic in which organizational identification is channeled into active creative contributions (Luu et al., 2019; Sánchez-Cardona et al., 2018). In summary, charismatic leadership amplifies the motivational impact of organizational identification through a constellation of micro-processes—including enhanced psychological safety, legitimacy provision, and creative self-efficacy—that together legitimize innovation as a form of identity expression and collective contribution.

Indeed, Feng et al. (2025) demonstrate that charismatic leadership moderates the effect of team stressors on resilience in China. Specifically, it enhances the positive impact of challenge stressors and mitigates the negative consequences of hindrance stressors on collective team identification, thereby boosting overall team resilience. G. Liu et al. (2015) studied employees in U.K. nonprofit organizations and found that charismatic leadership reinforces key connections in the internal branding process. It strengthens the relationship between brand orientation behavior and emotional brand attachment, further enhancing the effect of emotional attachment on staff service involvement.

In conclusion, charismatic leadership cultivates a motivational climate, fosters cognitive stimulation, and provides emotional reinforcement that enables organizational identification to manifest as innovative work behavior. Rather than directly shaping identification, charismatic leadership acts as a situational catalyst by activating and amplifying employees’ existing psychological attachment to the organization. By creating conditions that encourage risk-taking, creativity, and value alignment, charismatic leaders transform latent identification into purposeful, innovation-oriented behavior. In contrast, without charismatic leadership, even highly identified employees may lack the psychological safety, inspirational framing, or normative support needed to turn their identification into action. The motivational potential of organizational identification may remain dormant, leading to passive compliance rather than proactive innovation. Based on these considerations, we propose the following hypothesis:

**Hypothesis** **2:** *Charismatic leadership moderates the positive relationship between organizational identification and innovative work behavior, such that the relationship is stronger when charismatic leadership levels are higher.*

## 3. Methodology

### 3.1. Data Sources and Sample

This study empirically tested its hypotheses using data from the 2024 Comparative Survey on Perceptions of Public and Private Sector Employees in Korea, conducted by the Korea Institute of Public Administration (KIPA). Although the original survey targeted both public and private sector employees, this study focuses exclusively on public officials—specifically national and local government employees—making individual public servants the unit of analysis.

The KIPA survey aimed to provide a comprehensive understanding of employees’ perceptions and attitudes across various topics, including human resource practices, organizational behavior, and administrative culture. The questionnaire included a wide array of items measuring core constructs such as organizational identification, proactivity, leadership, decision-making autonomy, goal clarity, procedural and distributive justice, work–life balance, and diversity, equity, and inclusion. These variables establish a strong empirical foundation for examining major themes in public sector organizational behavior. This study specifically concentrates on innovative work behavior, goal clarity, and leadership—areas well-represented in the survey, ensuring content validity.

To ensure robust and representative sampling, the public sector sample was drawn from the 2023 National Census of Public Officials by the Ministry of Personnel Management. The final sample included 1000 individuals—500 national government officials and 500 local government officials. A proportional allocation method was applied, ensuring at least five respondents from each of the Grade 4-and-above and Grade 5 groups to secure sufficient representation across ranks.

The online survey was administered over a three-week period from 11 October to 1 November 2024, resulting in a final valid sample of 1012 public officials, with a 95% confidence level and a ±2.2% margin of error. The sampling design was stratified by key demographic and organizational factors, including level of government (central vs. local), gender, and rank. This rigorous approach enhances the generalizability of the study’s findings to the broader public sector workforce in Korea. The sociodemographic profile of the respondents included in this study is presented in Table 1.

### 3.2. Measures

#### 3.2.1. Innovative Work Behavior

The dependent variable in this study is innovative work behavior, referring to individuals’ proactive efforts to generate and implement new ideas that diverge from conventional methods to address work-related challenges and improve both organizational and individual performance (Janssen, 2000; Scott & Bruce, 1994). This concept encompasses more than just idea generation; it includes the creative process of transforming those ideas into tangible changes within the workplace. To measure innovative work behavior, this study utilized the following items: (1) “I try new ways of doing things to perform my job better,” (2) “I come up with new ideas to improve how I do my work,” and (3) “I attempt to introduce changes to key aspects of how my work is carried out.”

#### 3.2.2. Organizational Identification

Organizational identification was assessed by measuring the degree to which public employees psychologically associate themselves with their organization and internalize its successes and failures as their own (Carmeli & Freund, 2009; Yang & Mostafa, 2024). Six items were used to capture this construct, emphasizing emotional attachment, collective pride, and self-referential language. Respondents indicated their level of agreement with the statements: (1) “When I hear others criticize my organization, I feel like they are criticizing me,” (2) “I am very interested in what people think about my organization,” (3) “When talking about my organization, I often use ‘we’ rather than ‘the company’ or ‘the office,’”(4) “When my organization succeeds, I feel like I succeed,” (5) “When I hear others praise my organization, it feels like I’m being praised,” and (6) “When there is negative news about my organization, I feel embarrassed.”

#### 3.2.3. Charismatic Leadership

Charismatic leadership, the moderating variable in this study, is defined as a leadership style in which leaders articulate a compelling vision and sense of mission, demonstrate personal sacrifice and risk-taking to realize that vision, and inspire commitment and engagement among organizational members (Conger & Kanungo, 1998; Shamir & Howell, 1999). Charismatic leaders are distinguished by their exceptional insight and personal conviction, enabling them to effectively communicate organizational goals while maximizing collective performance through strong motivational influence. In public organizations, this leadership style is reflected in vision-setting, strategic acumen, and dedication to the organization—factors that enhance employees’ intrinsic motivation and public service motivation, ultimately fostering proactive behavior. Charismatic leadership in this study was measured using four items: (1) “My supervisor presents an ambitious strategy and clear goals for the organization” (vision clarification and strategic direction), (2) “My supervisor seizes new opportunities that help the organization achieve its goals” (environmental sensitivity and strategic insight), (3) “My supervisor is willing to make personal sacrifices and take risks for the benefit of the organization” (risk-taking and self-sacrifice), and (4) “My supervisor strives to realize a vision that is both ambitious and attainable” (vision execution and behavioral consistency).

#### 3.2.4. Controls

Several control variables were included to account for individual and organizational factors that could be associated with employees’ innovative work behavior. Gender was controlled (female = 1, male = 0) to capture potential differences in innovation-related attitudes or opportunities across sexes. Job rank was grouped into Grades 8–9, 6–7, and 1–5 to reflect hierarchical status, which may be linked to access to decision-making or innovation roles. Education was classified into five levels—high school or below, associate degree, bachelor’s degree, master’s degree, and doctoral degree—since higher education often correlates with increased cognitive flexibility and creativity. Age was divided into five brackets (under 30, 30s, 40s, 50s, and 60 and above) to account for generational differences in adaptability to change or technology. Tenure was categorized as 10 years or less, 20–29 years, and 30 years or more, recognizing that tenure can influence familiarity with institutional norms and the willingness to propose change. Finally, government level was included as a binary variable (local government = 1, central government = 0), as organizational structure and innovation culture may vary across administrative tiers.

### 3.3. Measurement Reliability and Validity

This study employed a total of 13 items to measure organizational identification, charismatic leadership, and innovative work behavior. Each item was rated on a five-point Likert scale ranging from 1 (“strongly disagree”) to 5 (“strongly agree”). To evaluate the construct validity of the survey instrument, an exploratory factor analysis (EFA) was conducted. Prior to factor extraction, the Kaiser–Meyer–Olkin (KMO) measure of sampling adequacy was calculated to determine the appropriateness of the data for factor analysis. The overall KMO values for each latent variable exceeded the commonly accepted threshold of 0.70, indicating that the data were suitable for factor analysis.

The criteria for factor extraction were set at eigenvalues greater than or equal to 1.0, and only items with factor loadings of 0.5 or higher were retained. An orthogonal varimax rotation was applied to enhance the interpretability of the factor structure. The analysis identified three distinct latent factors, with all items loading cleanly onto their respective factors. Every item demonstrated a factor loading of 0.5 or higher, confirming the construct validity of the measurement scales. Additionally, reliability analysis indicated satisfactory internal consistency for each factor: Cronbach’s α was 0.828 for organizational identification, 0.893 for charismatic leadership, and 0.849 for innovative work behavior. These results suggest that the measurement items exhibited strong reliability, exceeding the commonly accepted threshold of 0.7. Detailed results of the factor and reliability analyses are presented in Table 2.

## 4. Results

Table 3 presents descriptive statistics and correlation coefficients for the key variables in this study, particularly focusing on the dependent variable, innovative work behavior. The analysis shows that organizational identification is significantly and positively correlated with innovative work behavior (*r* = 0.352, *p* < 0.01), suggesting that employees who feel a strong psychological bond with their organization are more likely to engage in discretionary and creative efforts aimed at improvement. Charismatic leadership also shows a meaningful positive correlation with innovative behavior (*r* = 0.256, *p* < 0.01), implying that leadership style may play an enabling role in motivating innovation. Among the control variables, tenure (*r* = 0.228, *p* < 0.01), age (*r* = 0.231, *p* < 0.01), job grade (*r* = 0.175, *p* < 0.01), and education (*r* = 0.129, *p* < 0.01) all show small but significant positive correlations with innovative work behavior, while gender (*r* = −0.071, *p* < 0.01) shows a weak negative correlation. Government level is not significantly correlated with innovative behavior.

To establish a baseline model, Model 1 was developed to evaluate the impact of demographic and organizational control variables on innovative work behavior, alongside the direct influence of charismatic leadership. This initial analysis provides insight into how leadership style and individual characteristics relate to employees’ willingness to innovate. Model 2 introduced the primary independent variable, organizational identification, to determine whether employees’ psychological attachment to their organization contributes to innovative work behavior beyond the effects of leadership and demographic factors. By including this variable, we were able to more accurately estimate the unique contribution of organizational identification. Finally, Model 3 integrated the interaction term between organizational identification and charismatic leadership to test the proposed moderating effect, examining whether the relationship between organizational identification and innovative work behavior varies based on the level of charismatic leadership perceived by employees.

The results from Model 1 in Table 4 indicate that among the control variables, both education (β = 0.096, *p* < 0.01) and age (β = 0.117, *p* < 0.01) have a significant positive association with innovative work behavior. This suggests that more educated and older employees are more likely to engage in innovation, possibly due to accumulated knowledge, confidence, or experience that enables them to pursue nonroutine work behaviors. Charismatic leadership also has a strong positive effect on innovative work behavior (β = 0.192, *p* < 0.001), indicating that employees are more likely to behave innovatively when led by leaders who are inspirational and vision-driven.

In Model 2, the introduction of the independent variable, organizational identification, reveals a significant positive relationship with innovative work behavior (β = 0.258, *p* < 0.001). This finding supports Hypothesis 1 that employees who identify strongly with their organization are more likely to go beyond routine responsibilities to contribute creatively. The coefficient for charismatic leadership remains significant (β = 0.119, *p* < 0.001), further emphasizing its relevance as a direct driver of innovative work behavior.

Model 3 introduces the interaction term between organizational identification and charismatic leadership to test the moderating effect. The interaction term is statistically significant (β = 0.060, *p* < 0.01), suggesting that charismatic leadership strengthens the positive relationship between organizational identification and innovative work behavior. In other words, the influence of organizational identification on innovation is more pronounced when employees perceive their leaders to be charismatic. This result supports Hypothesis 2 that charismatic leadership functions as a contextual enhancer that activates employees’ psychological commitment to innovative action.

**Figure 2:**
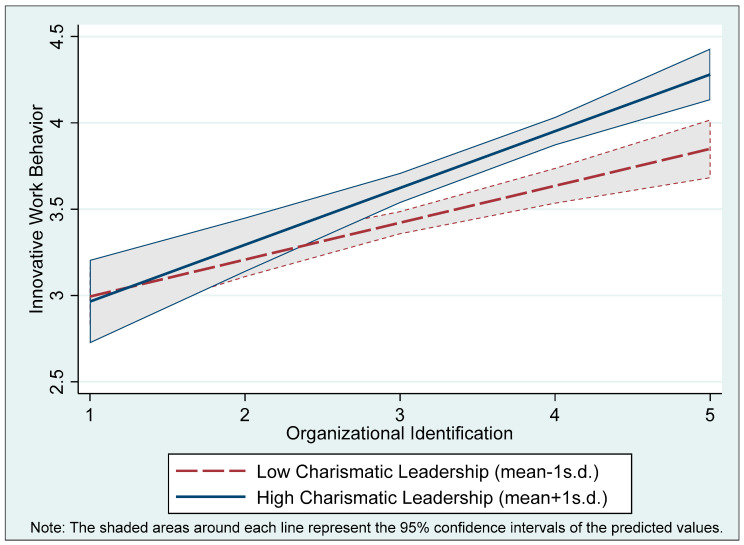
Moderating effect of charismatic leadership on the relationship between organizational identification and innovative work behavior.

Figure 2 visually illustrates how the relationship between organizational identification and innovative work behavior varies with different levels of charismatic leadership. When charismatic leadership is high (mean + 1 standard deviation), the positive association between organizational identification and innovative work behavior becomes more pronounced. As employees’ organizational identification increases, their level of innovative behavior rises more sharply under high-charisma conditions. Conversely, when charismatic leadership is low (mean − 1 standard deviation), the slope of the relationship appears more modest, suggesting a weaker tendency. This pattern implies that charismatic leadership may amplify the psychological mechanisms through which organizational identification relates to innovative work behavior. Although the 95% confidence intervals partially overlap, the divergence between the two lines becomes more evident—particularly when organizational identification exceeds the scale midpoint—indicating that the moderating effect is likely more pronounced at higher levels of identification.

These findings support the interpretation that charismatic leadership functions as a contextual amplifier, enhancing the psychological conditions under which organizational identification translates into innovative work behavior. Charismatic leaders provide emotional framing, intellectual stimulation, and value alignment that activate and magnify the motivational power of identification. When employees feel a strong sense of belonging to the organization, the presence of a visionary and supportive leader increases their confidence to challenge norms, take risks, and pursue novel ideas in the service of collective goals. In such environments, the synergy between identification and charismatic leadership fosters forward-looking, change-oriented behavior. Conversely, in the absence of charismatic leadership, even highly identified employees may lack the psychological safety or inspirational guidance needed to express their attachment through innovation. Thus, charismatic leadership creates the conditions that enable latent organizational identification to manifest as active, creative contributions to organizational progress.

## 5. Discussion

### 5.1. Theoretical Implications

This study contributes to the literature on behavioral public administration by integrating social identity theory and charismatic leadership theory, providing a more comprehensive understanding of how innovative work behavior arises in public organizations. Traditionally, research on public sector innovation has focused on either intrinsic motivational factors—such as public service motivation or organizational commitment—or on leadership styles that inspire employees to act beyond their formal roles. However, few studies have examined how employee identification and leadership interact to drive innovation. This research addresses that gap by offering both theoretical integration and empirical evidence that demonstrates the effects of organizational identification are significantly shaped by the leadership context.

First, this study extends social identity theory by demonstrating that organizational identification serves as a potent source of intrinsic motivation for innovation. When employees internalize their organization’s mission and values as part of their self-concept, they are more inclined to engage in discretionary behaviors aimed at improving organizational performance. This internalized identity creates a personal stake in the organization’s success, motivating individuals to act proactively, even in environments where external rewards are limited. While prior research has primarily focused on identification’s role in promoting organizational commitment or job satisfaction, this study empirically verifies its relevance to innovation, broadening the applicability of social identity theory within public sector contexts.

Second, this study advances charismatic leadership theory by reframing it as a contextual mechanism of identity activation rather than merely another moderator. Its impact is not uniform; in bureaucratic settings, organizational identification alone does not automatically produce innovation. Instead, innovation occurs when leaders create legitimacy and psychological safety, enabling employees to act on their identity without fear of sanction or failure. Charismatic behaviors—such as articulating a compelling vision, modeling shared values, and symbolically reinforcing group identity—serve as triggers that activate identification into innovative behavior. In doing so, the study contributes to social identity theory by showing that identity-based motivation is conditional rather than automatic, and supports a multiplicative model of employee-driven innovation where leadership interventions are most effective when they activate and channel pre-existing psychological resources.

Finally, this study advances theoretical discourse by moving beyond structural or institutional explanations of public sector innovation, which often emphasize resource availability, regulatory reform, or managerial discretion. Instead, it highlights psychological attachment and leadership framing as internal drivers for innovation, even in constrained environments. This perspective is particularly relevant for public organizations that operate under rigid procedures and hierarchical controls, where formal incentives for innovation may be limited. By focusing on the motivational and symbolic mechanisms that stimulate innovation, the study provides a compelling alternative to purely structural explanations and underscores the importance of behavioral insights in understanding public administration.

### 5.2. Practical Implications

To promote innovative work behavior in public organizations, it is essential to adopt a human resource strategy that strengthens employees’ psychological attachment to the organization and fosters value-based leadership. While many public sector reforms prioritize structural changes or performance incentives, this study shows that innovation is more effectively nurtured by enhancing two key internal drivers: organizational identification and charismatic leadership. When supported by a cohesive set of HR practices, these factors can create a work environment conducive to creativity, risk-taking, and commitment to public service.

First, value-based recruitment and early socialization are crucial for fostering strong organizational identification. Public agencies should highlight their mission and core values during the hiring process to attract candidates whose personal values align with those of the organization. This value congruence facilitates the internalization of public goals and fosters a sense of belonging from the outset. Supervisors who embody these values are more likely to emerge as inspirational leaders who motivate and mobilize their teams toward innovation.

Second, leadership development programs should intentionally cultivate traits associated with purpose-driven leadership, such as vision articulation, value-based communication, and emotional resonance. These leaders help translate identification into action by reinforcing shared purpose and creating psychological safety. Leadership training should prioritize ethical reasoning and symbolic behavior alongside administrative competence.

Third, performance management and organizational culture must reinforce innovation-oriented behaviors. Evaluation systems should recognize not only task efficiency but also contributions to learning, ethical conduct, and alignment with public values. A supportive culture that normalizes experimentation and celebrates success stories can further sustain innovation. Initiatives like innovation labs and idea-sharing platforms can institutionalize creative behavior and reduce the fear of failure.

However, organizations should also exercise caution. While inspirational leadership can strengthen purpose and engagement, it may also produce unintended side effects, particularly when it takes the form of charismatic authority concentrated in a single figure. Excessive identification with such leaders can foster psychological dependency, diminish critical thinking, and lead to hero worship that undermines collective accountability. These risks are especially pronounced in hierarchical or opaque organizations. To mitigate them, HR strategies should be paired with institutional checks, transparent decision-making, and participatory governance mechanisms that promote distributed leadership and organizational integrity.

By aligning recruitment, leadership development, and performance systems within a cohesive framework, public organizations can create a psychologically engaging environment that encourages innovation while maintaining ethical accountability.

### 5.3. Limitations and Future Research

Despite offering novel insights into the interaction between organizational identification and charismatic leadership in shaping innovative work behavior, this study has several limitations that warrant consideration and open avenues for future research.

First, the cross-sectional nature of the data means that the findings should not be interpreted as evidence of causality. While the results demonstrate a statistically significant interaction between organizational identification and charismatic leadership regarding innovative work behavior, they reflect associations rather than causal effects. The direction of influence remains uncertain, and it is plausible that reciprocal or third-variable relationships exist. Moreover, both organizational identification and innovative work behavior are dynamic processes that evolve over time in response to organizational change, leadership transitions, or fluctuations in institutional trust. Future research should adopt longitudinal or experimental designs capable of disentangling the temporal and causal ordering of leadership behavior, identification, and innovation.

Second, the empirical analysis is based exclusively on data from South Korean public organizations, raising concerns about the generalizability of the findings. Given the hierarchical and collectivist nature of Korean administrative culture, the strength of organizational identification and the influence of charismatic leadership may manifest differently in other cultural or institutional settings. Comparative studies across diverse national or sectoral contexts would help clarify the boundary conditions of the proposed relationships.

Third, although this study employed context-relevant items to measure charismatic leadership, it did not use the complete instrument developed by Conger and Kanungo (1998), one of the most widely validated scales in the literature. This limitation arose because the survey data were not originally designed to specifically evaluate charismatic leadership but rather to assess general features of organizational management in the public sector. Consequently, only a limited set of items capturing key aspects of charismatic leadership—such as vision clarity, strategic awareness, and consistent, value-driven behavior—were available for analysis. While these items capture essential aspects of charismatic leadership, they do not encompass the full conceptual range of the original scale. Therefore, caution is warranted in interpreting the construct’s validity and comparing the results with studies that used the complete Conger and Kanungo instrument. Future research should consider employing validated, multidimensional measures that more comprehensively reflect the rhetorical, behavioral, and affective dimensions of charismatic leadership.

Fourth, this study conceptualized organizational identification as a relatively stable psychological state. However, identification is likely to evolve over time, influenced by organizational changes, leadership transitions, or shifts in institutional trust. Future research should explore the dynamic nature of identification and its interaction with leadership over time. Such an approach could yield deeper insights into how identity-based mechanisms contribute to sustained innovative work behavior in public organizations.

Fifth, the findings of this study should be interpreted in light of the unique cultural characteristics of the South Korean public sector, which is characterized by strong hierarchical structures and collectivist norms. These features may have amplified the effects of organizational identification and value-based leadership. In such environments, employees are more likely to internalize collective goals, respond sensitively to leadership cues, and perceive innovation as a contribution to group success. Consequently, the observed relationships may differ in more individualistic or egalitarian public sectors, where autonomy and decentralized authority shape distinct motivational dynamics. Future research should examine whether these findings hold across diverse cultural and institutional contexts to better establish the generalizability of the results.

Sixth, while this study emphasizes the positive role of charismatic leadership and organizational identification in promoting innovative work behavior, it also acknowledges the potential downsides of their interaction. In highly hierarchical settings like the Korean public sector, strong identification with a charismatic leader can unintentionally foster conformity, suppress dissent, and encourage hero worship, particularly when emotional appeals override critical evaluation. These unintended consequences highlight the need to approach charismatic leadership not only as a catalyst for innovation but also as a double-edged sword that may reinforce existing power structures or discourage deviance when it threatens group cohesion. Future research should examine this “dark side” more systematically by incorporating measures of dissent tolerance, critical voice, or blind obedience, and by exploring the contextual moderators—such as psychological safety, participatory culture, or leader accountability—that shape whether charisma-activated identification results in constructive or maladaptive outcomes (X. Zhang et al., 2020). Such balanced theorization would contribute to a more nuanced understanding of when and how charismatic leadership enables identity-driven innovation without compromising organizational reflexivity or ethical vigilance.

Finally, future research would benefit from employing multilevel designs to capture the collective dynamics of identification and leadership climate. While this study focused on individual-level perceptions, organizational identification and leadership are often shared experiences shaped by team norms and agency-wide cultures. Analyzing group- or agency-level effects would help illuminate how shared identity and emotionally resonant leadership collectively influence innovation. This approach would not only enhance explanatory power but also provide insights into how contextual and structural features shape identity-based mechanisms in public organizations.

### 5.4. Conclusions

This study prompts a reevaluation of the concept of innovation within public organizations, suggesting it should not be viewed solely as a result of structural changes or external pressures. Instead, it emerges as a more viable phenomenon when employees feel a psychological attachment and are led by figures who provide purpose, coherence, and symbolic direction. As indicated in Table 5, the findings validate both hypotheses: organizational identification is positively linked to innovative work behavior, and this connection is strengthened under conditions of charismatic leadership. These results underscore the importance of alignment between employees’ internalized sense of belonging and the symbolic messages they receive from their leaders. While such alignment does not guarantee innovation, it fosters an environment conducive to proactive and constructive behaviors.

Notably, this study contributes to the broader field of behavioral public administration by emphasizing how psychological and relational mechanisms—such as identity, emotional resonance, and leadership symbolism—drive innovation within bureaucratic systems. By moving beyond purely structural or procedural explanations, it adds to the growing body of research that foregrounds employee cognition, motivation, and social context in shaping public sector outcomes.

Situated within the Korean public sector, this study further illustrates how national and institutional contexts can shape the operation of these mechanisms. In an environment characterized by strong hierarchies and collectivist norms, charismatic leadership may be especially potent in mobilizing organizational identification into proactive behavior. Far from limiting generalizability, the Korean case provides a theoretically meaningful boundary condition that helps explain when and why value-based leadership enhances innovation in bureaucracies.

For public agencies facing increasing complexity and accountability, this perspective implies that nurturing innovation may rely more on developing internal relational conditions than on implementing external structural reforms. Additionally, it encourages future research into how relational mechanisms—like trust, fairness, and value congruence—interact with leadership to influence employee behavior in changing institutional contexts.

## Figures and Tables

**Table 1:** Demographic information on the sample.

Category	Subcategory	Number of Cases	Percentage (%)
Gender	Female	456	45.06
	Male	556	54.94
Job Grade	Grades 8–9	315	31.13
	Grades 6–7	536	52.06
	Grades 1–5	161	15.91
Education	High school or lower	27	2.67
	Associate degree	26	2.57
	Bachelor’s degree	702	69.37
	Master’s degree	195	19.27
	Doctoral degree	62	6.13
Age	20s or younger	182	9.01
	30s	662	32.77
	40s	687	34.01
	50s	421	20.84
	60s or older	68	3.37
Tenure	10 years or less	472	46.64
	11–20 years	371	36.66
	Over 30 years	169	16.70
Government level	Central government	503	49.70
	Local government	509	50.30

**Table 2:** Factor loadings, Cronbach’s α, and KMO.

Latent Variables	Survey Items	Factor 1	Factor 2	Factor 3	Cronbach’s α	KMO
Innovative Work Behavior	- I try new ways of doing things to perform my job better.	0.847			0.849	0.840
	- I come up with new ideas to improve how I do my work.	0.872				0.801
	- I attempt to introduce changes to key aspects of how my work is carried out.	0.857				0.817
Organizational Identification	- When I hear others criticize my organization, I feel like they are criticizing me.		0.745		0.828	0.897
	- I am very interested in what people think about my organization.		0.668			0.935
	- When talking about my organization, I often use ‘we’ rather than ‘the company’ or ‘the office.’		0.649			0.938
	- When my organization succeeds, I feel like I succeed.		0.691			0.873
	- When I hear others praise my organization, it feels like I’m being praised.		0.783			0.857
	- When there is negative news about my organization, I feel embarrassed.		0.653			0.923
Charismatic Leadership	- My supervisor presents an ambitious strategy and clear goals for the organization.			0.857	0.893	0.885
	- My supervisor seizes new opportunities that help the organization achieve its goals.			0.853		0.888
	- My supervisor is willing to make personal sacrifices and take risks for the benefit of the organization.			0.832		0.902
	- My supervisor strives to realize a vision that is both ambitious and attainable.			0.855		0.886

**Table 3:** Descriptive statistics and correlations.

	(1)	(2)	(3)	(4)	(5)	(6)	(7)	(8)	(9)
(1)	1								
(2)	0.352 ***	1							
(3)	0.256 ***	0.380 ***	1						
(4)	−0.071 **	−0.062 **	−0.088 ***	1					
(5)	0.175 ***	0.252 ***	0.125 *	−0.115 ***	1				
(6)	0.129 ***	0.091 ***	0.041	−0.044	0.188 ***	1			
(7)	0.231 ***	0.290 ***	0.093 *	−0.223 ***	0.495 ***	0.186 ***	1		
(8)	0.228 *	0.282 ***	0.143	−0.180 ***	0.537 ***	0.134 ***	0.766 ***	1	
(9)	−0.027	0.012	−0.078 **	0.134 ***	−0.108 ***	−0.136 ***	0.027	0.013	1
mean	3.607	3.291	2.954	0.451	1.848	3.236	2.528	1.762	0.503
s.d.	0.853	0.825	1.004	0.498	0.669	0.718	0.905	0.799	0.500

Note: * *p* < 0.1; ** *p* < 0.05; *** *p* < 0.01; (1) = Innovative Work Behavior; (2) = Organizational Identification; (3) = Charismatic Leadership; (4) = Gender; (5) = Job Grade; (6) = Education; (7) = Age; (8) = Tenure; (9) = Government level; s.d. = standard deviation.

**Table 4:** Hierarchical multiple linear regression models for the hypothesized relationships.

	Model 1		Model 2		Model 3	
	β (S.E.)		β (S.E.)		β (S.E.)	
Gender	−0.005		−0.014		−0.012	
	(0.053)		(0.051)		(0.051)	
Job Grade	0.040		0.007		0.002	
	(0.046)		(0.045)		(0.045)	
Education	0.096	***	0.088	**	0.089	**
	(0.036)		(0.035)		(0.035)	
Age	0.117	***	0.077	*	0.077	*
	(0.045)		(0.044)		(0.044)	
Tenure	0.077		0.064		0.063	
	(0.052)		(0.050)		(0.050)	
Government Level	0.001		−0.018		−0.02	
	(0.052)		(0.051)		(0.051)	
Charismatic Leadership	0.192	***	0.119	***	−0.075	
	(0.026)		(0.027)		(0.090)	
Organizational Identification			0.258	***	0.087	
			(0.034)		(0.082)	
Organizational Identification × Charismatic Leadership					0.060	**
					(0.027)	
Constant	2.224	***	1.816	***	2.363	***
	(0.157)		(0.162)		(0.290)	
$R^{2}$	0.119		0.167		0.172	

Note: * *p* < 0.1; ** *p* < 0.05; *** *p* < 0.01; S.E. = robust standard error.

**Table 5:** Hypothesis testing results.

Hypothesis	Result
**Hypothesis 1:** *Organizational identification is positively associated with innovative work behavior among public sector employees.*	Supported
**Hypothesis 2:** *Charismatic leadership moderates the positive relationship between organizational identification and innovative work behavior, such that the relationship is stronger when charismatic leadership levels are higher.*	Supported

## Data Availability

The data used for this study are available at https://www.kipa.re.kr/html/kor/stt/stat/statDbTab.do. Permission to use data must be obtained from KIPA. (accessed on 6 April 2025).
